# A Proposed Convergent Genetic and Molecular Framework for Legg–Calvé–Perthes Disease: A Scoping Review

**DOI:** 10.3390/ijms27198890

**Published:** 2026-10-07

**Authors:** Edgar Hernández-Zamora, Armando Odiseo Rodríguez-Olivas, Marlene Alejandra Galicia-Alvarado, Leonora Casas-Ávila, Elba Reyes-Maldonado

**Affiliations:** 1 Genomic Medicine, Instituto Nacional de Rehabilitación “Luis Guillermo Ibarra Ibarra”, Calzada México-Xochimilco 289, Arenal de Guadalupe, Tlalpan, Mexico City 14389, Mexico; 2 Instituto de Ciencias Biomédicas, Universidad Autónoma de Ciudad Juárez, Campus Cuauhtémoc, Km. 3.5 Carretera Anáhuac s/n, Anáhuac, Municipio de Cuauhtémoc, Chihuahua 31600, Mexico; 3 Neurociencias Clínicas, Instituto Nacional de Rehabilitación “Luis Guillermo Ibarra Ibarra”, Calzada México-Xochimilco 289, Arenal de Guadalupe, Tlalpan, Mexico City 14389, Mexico; 4 Morphology Department, Escuela Nacional de Ciencias Biológicas, Instituto Politécnico Nacional, Prolongación de Carpio y Plan de Ayala s/n, Santo Tomás, Gustavo A. Madero, Mexico City 11340, Mexico

**Keywords:** Legg–Calvé–Perthes disease, genetic variants, epigenetics, inflammation, coagulation, bone remodeling

## Abstract

Legg–Calvé–Perthes disease (LCPD) is a rare pediatric disorder characterized by avascular osteonecrosis of the femoral head. Its pathogenesis is multifactorial, and genetic, epigenetic, vascular, and inflammatory mechanisms may contribute to disease susceptibility and progression. To map and characterize the available evidence on genetic and epigenetic factors and molecular pathways associated with LCPD, with particular emphasis on potential interactions and convergence across biological pathways. A scoping review was conducted in accordance with the Preferred Reporting Items for Systematic Reviews and Meta-Analyses Extension for Scoping Reviews (PRISMA-ScR, PRISMA 2020 for Abstracts checklist). PubMed/MEDLINE was searched for studies published between March 2006 and March 2026. Relevant genetic, epigenetic, and molecular findings were extracted and synthesized according to biological pathways. See the PRISMA 2020 for Abstracts checklist. Seventeen genes and 25 single-nucleotide variants (SNVs), as reported in the included literature, were identified and grouped into three major biological axes: hypercoagulability, inflammation, and bone metabolism and remodeling. Associations with LCPD were reported in at least one study for *F5* rs6025, *MTHFR* rs1801133, *IL6* rs1800795, *IL23R* rs1569922, *NOS3* rs1799983 and rs2070744, and *OPG* rs2073618. Epigenetic mechanisms, including DNA methylation and microRNA dysregulation, were also reported. Interpretation was limited by methodological heterogeneity, small study populations, limited functional validation, and a predominance of observational studies. Accumulating evidence suggests that thrombotic, inflammatory, bone remodeling, and epigenetic mechanisms contribute to LCPD susceptibility and pathogenesis. Our findings point to a potential biological convergence among these pathways; however, whether this convergence stems from a shared genetic architecture remains to be fully elucidated. LCPD pathogenesis involves a complex interplay of coagulation, inflammation, bone remodeling, and epigenetic pathways. While these mechanisms suggest biological convergence, their precise shared genetic architecture remains uncertain. Consequently, future studies utilizing independent replication and functional validation are critically needed.

## 1. Introduction

Rare diseases comprise a heterogeneous group of conditions in which genetic factors may contribute to disease susceptibility, clinical variability, and pathogenesis [1]. Genetic architecture refers to the combination of genetic variants and their interactions with environmental and biological factors that influence disease-related phenotypes [2,3,4,5]. In multifactorial disorders, individual variants may exert modest effects and interact with other genetic or environmental factors.

Legg–Calvé–Perthes disease (LCPD) is a rare pediatric disorder with an estimated worldwide prevalence ranging from 0.4 to 29 cases per 100,000 individuals [1]. It is characterized by avascular osteonecrosis of the femoral head (FH), resulting in hip pain, limited joint mobility, and gait disturbances. LCPD occurs more frequently in boys than in girls and typically presents between 4 and 8 years of age.

During the early stage of LCPD, the FH may appear radiographically normal or exhibit a small sclerotic epiphysis accompanied by widening of the joint space. This is followed by an ischemic phase characterized by interruption of the blood supply to the femoral epiphysis, leading to osteonecrosis. Subsequently, reparative processes involving revascularization and resorption of necrotic bone and cartilage are initiated. However, mechanical loading of the hip during this stage may result in collapse of the FH and the development of characteristic deformities, including coxa plana and coxa vara. Histopathological findings include degeneration of the articular cartilage, trabecular bone necrosis, fibrovascular tissue infiltration, and increased osteoclastic bone resorption (Figure 1) [6,7,8,9].

The diagnosis of LCPD is primarily based on anteroposterior and frog-leg lateral radiographs, which remain the gold standard for imaging evaluation, although radiographic abnormalities may be subtle during the early stages of the disease. Radiographically, LCPD progresses through four sequential stages: necrosis, fragmentation, reossification, and remodeling (healing). This process may extend over several years and ultimately result in varying degrees of femoral head (FH) deformity [10,11,12].

Treatment options include both conservative and surgical approaches. Conservative management aims to preserve hip range of motion, reduce mechanical loading of the joint, and maintain femoral head containment, often in combination with analgesic and anti-inflammatory medications. Surgical intervention is generally recommended when containment cannot be maintained, particularly in children older than six years. Among the available procedures, acetabular reorientation techniques, including triple pelvic osteotomy, are increasingly used to improve femoral head coverage. The principal prognostic factors include the patient’s age at diagnosis, hip range of motion, and the extent of FH involvement according to the Herring lateral pillar and Catterall classifications (Figure 2) [12,13].

Although the etiology of LCPD remains incompletely understood, accumulating evidence supports a significant genetic contribution to disease susceptibility. Familial aggregation and the identification of potential inheritance patterns suggest that both pathogenic variants and shared genetic polymorphisms may increase the risk of developing LCPD across different populations [14,15,16,17,18].

Current evidence indicates that LCPD results from the interaction of multiple biological mechanisms rather than a single pathogenic process. Hypercoagulable states, inherited thrombophilia, and impaired fibrinolysis may promote vascular occlusion, compromise blood supply to the FH, and ultimately lead to ischemic osteonecrosis. Genetic alterations affecting bone matrix integrity, particularly variants in *COL2A1* and *COL1A1*, have also been implicated. Furthermore, abnormal bone repair appears to result from an imbalance between osteoclastic bone resorption and osteoblastic bone formation, mediated in part by pro-inflammatory and pro-osteoclastogenic cytokines, including tumor necrosis factor-α (TNF-α), interleukin (IL)-1, and IL-6. More recently, chronic inflammation has emerged as an additional pathogenic mechanism, with elevated inflammatory mediators and systemic biomarkers, such as the neutrophil-to-lymphocyte ratio, being associated with disease severity and clinical progression [19,20,21,22].

In this review, we propose a convergent framework that integrates coagulation, inflammatory, and bone remodeling pathways in response to vascular alterations. This model also incorporates biomarkers of bone metabolism and structural integrity, together with environmental influences, providing a comprehensive framework for understanding the complex pathogenesis of LCPD.

Building on this conceptual framework, the present scoping review examines the available evidence on the genetic architecture of LCPD, with particular emphasis on the interplay among coagulation, inflammation, bone metabolism, and genetic susceptibility [23,24,25].

The proposed framework is intended to organize the available evidence and generate mechanistic hypotheses; it does not imply that causal relationships among the proposed pathways have been established.

### 1.1. Research Question

This study aims to answer the following research questions: what evidence exists regarding the genetic factors and molecular pathways associated with susceptibility to and the pathogenesis of Legg–Calvé–Perthes disease, how have these mechanisms been characterized in human and preclinical studies, and what evidence supports their convergence across biological pathways toward a shared genetic architecture of disease susceptibility?

### 1.2. Objective

This study aims to map and characterize the available evidence on genetic and epigenetic factors and molecular pathways associated with susceptibility to and the pathogenesis of LCPD, with particular emphasis on their potential convergence across biological pathways.

## 2. Methods

### 2.1. Study Design and Reporting Framework

This study was conducted as a scoping review of the literature and was reported in accordance with the Preferred Reporting Items for Systematic Reviews and Meta-Analyses 2020 extension for Scoping Reviews (PRISMA-ScR). The review was designed to map and characterize the available evidence on genetic and epigenetic factors and molecular pathways associated with susceptibility to and the pathogenesis of Legg–Calvé–Perthes disease (LCPD) across human and preclinical studies, and to identify patterns, inconsistencies, and gaps in the existing literature.

### 2.2. Eligibility Framework

Based on the review question, the eligibility framework was structured according to the Population–Concept–Context (PCC) model (Table 1).

### 2.3. Information Sources

A systematic search of PubMed/MEDLINE was conducted for studies published from March 2006 through March 2026, representing a 20-year search period. No language restrictions were applied. Additional records were identified through reference-list screening and other relevant sources. Two web-based sources were used exclusively to provide epidemiological or contextual information and were not considered scientific evidence sources.

### 2.4. Search Strategy

The search strategy combined terms related to Legg–Calvé–Perthes disease with terms related to etiology, genetic and epigenetic mechanisms, polymorphisms, coagulation, inflammation, syndromic associations, and mutations. The PubMed/MEDLINE search strategy was:

(“Perthes Disease”[Mesh] OR “Legg-Calvé-Perthes Disease”[tiab] OR “Legg Calve Perthes”[tiab] OR “Perthes disease”[tiab] OR LCPD[tiab])

AND

(etiology[tiab] OR etiology[Mesh] OR genetic*[tiab] OR “Genetic Predisposition to Disease”[Mesh] OR “Polymorphism, Single Nucleotide”[Mesh] OR polymorphism*[tiab] OR mutation*[tiab] OR epigenetic*[tiab] OR methylation[tiab] OR microRNA*[tiab] OR coagulation[tiab] OR thrombophilia[tiab] OR hypercoagulab*[tiab] OR inflammation[tiab] OR inflammatory[tiab] OR syndrome*[tiab])

AND

(“2006/03/01”[Date—Publication]: “2026/03/31”[Date—Publication])

### 2.5. Eligibility Criteria

The PCC framework was used to define the eligibility criteria. The population comprised individuals with LCPD and relevant preclinical models. The concept included genetic and epigenetic factors and molecular pathways potentially involved in disease susceptibility or pathogenesis. The context encompassed studies addressing vascular, thrombotic, inflammatory, metabolic, structural, epigenetic, and syndromic mechanisms related to LCPD.

Original research studies, familial studies, case reports with relevant genetic or molecular findings, and relevant review articles were considered. Eligible studies were required to provide evidence directly related to LCPD. Studies addressing molecular or biological mechanisms in other diseases without direct evidence relevant to LCPD were considered contextual references rather than direct evidence sources. The reviews were used as sources for mapping or contextualization, but not every review was treated as independent primary evidence.

Studies were excluded when they focused exclusively on clinical management, surgery, rehabilitation, imaging, or prognosis without a genetic or molecular component; addressed other forms of osteonecrosis without a specific relationship to LCPD; or consisted of editorials, commentaries, letters without original data, protocols, conference abstracts without sufficient information, or duplicate publications.

### 2.6. Selection of Sources of Evidence

Two reviewers independently screened all identified records in duplicate according to the predefined eligibility criteria and the PCC framework. The selection process was conducted in two sequential stages. First, titles and abstracts were independently screened to identify records potentially relevant to the review question. Second, the full texts of potentially eligible reports were independently assessed by both reviewers to determine final eligibility. Disagreements at either stage were resolved through discussion and consensus. Reasons for exclusion at the full-text stage were recorded.

The PubMed/MEDLINE search identified 2583 records, and six additional records were identified through other sources. After removal of duplicate records, 1113 records remained for title and abstract screening. Following title and abstract screening, 190 reports were sought for retrieval. Of these, 84 reports could not be retrieved, leaving 106 reports for full-text assessment. After full-text assessment, 22 reports were excluded because they did not meet the predefined eligibility criteria. The final selection comprised 84 scientific publications. Two additional web-based sources were retained exclusively for epidemiological and contextual information.

The selection process and the numbers of records identified, screened, assessed for eligibility, excluded, and included are presented in the PRISMA 2020 flow diagram (Figure 3).

### 2.7. Protocol and Registration

A review protocol was not prospectively registered. The review was conducted and reported in accordance with the PRISMA-ScR reporting framework. The eligibility criteria and review question were defined before the selection of sources.

## 3. Results

A total of 84 scientific publications were included in the qualitative synthesis, comprising 44 original research articles and 40 review articles. Original research articles were considered the primary source of direct evidence, whereas review articles were used for evidence mapping and contextualization. Two additional web-based sources were retained exclusively for epidemiological or contextual information and were not considered direct scientific evidence (Figure 3).

The results were synthesized qualitatively and narratively because the included evidence was heterogeneous in terms of study populations, genetic markers, outcome definitions, and analytical approaches. This approach was considered appropriate for mapping the breadth and characteristics of the available evidence rather than estimating a pooled effect. No meta-analysis was performed; therefore, no statistical model, formal assessment of statistical heterogeneity, or meta-analysis software was used.

In general, the strength of the available evidence was limited by substantial methodological heterogeneity, small study populations, limited functional validation, and the pre-dominance of observational and case-based studies. Table 2 includes seventeen genes and 25 single-nucleotide variants (SNVs) reported in patients with LCPD, organized according to three major pathophysiological axes: hypercoagulability, pro-inflammatory responses, and bone metabolism and structure.

Overall, the available evidence suggests that the majority of the genetic variants investigated have not demonstrated a consistent association with LCPD. Epigenetic mechanisms, including DNA methylation and microRNA dysregulation, were also implicated. However, these findings should be interpreted in the context of the study population, sample size, analytical model, and degree of independent replication. Nevertheless, the variants *FVL* rs6025, *MTHFR* rs1801133, *IL-6* rs1800795, *IL-23R* rs1569922, *NOS3* rs1799983, *NOS3* rs2070744, and *OPG* rs2073618 have been reported to be positively associated with LCPD in at least one study, suggesting that thrombophilia, inflammatory, and bone remodeling pathways may contribute to the pathophysiology of LCPD. The available evidence does not establish that any of these variants independently determines LCPD susceptibility.

## 4. Discussion

Although the precise mechanisms underlying femoral head osteonecrosis in LCPD remain incompletely understood, the available literature suggests that genetic and molecular factors may influence vascular, inflammatory, and bone remodeling processes. In this review, we propose a convergent framework comprising three interconnected axes: hypercoagulability, pro-inflammatory responses, and bone metabolism and structure. This framework is intended to integrate the available evidence and identify potential mechanistic relationships rather than to establish causal pathways.

### 4.1. Hypercoagulable States Axis

Several studies suggest that hypercoagulable or prothrombotic states (thrombophilia) contribute to the pathogenesis of LCPD. Genetic factors play a crucial role in this process, as certain variants promote a prothrombotic imbalance that increases the risk of clot formation. These alterations may contribute to a prothrombotic state and could be involved in the vascular mechanisms proposed in the pathogenesis of LCPD; however, their causal role in the disease remains unestablished.

In genomics, single-nucleotide polymorphisms (SNPs) are common (≥1% frequency), whereas SNVs, term used in recent years, include all such variants regardless of frequency. The SNV, *PT* rs1799963, also known as the G20210A mutation in the prothrombin gene, is a well-recognized inherited risk factor for thrombophilia. This variant consists of a guanine (G) to adenine (A) substitution in the 3′ untranslated region and is associated with increased circulating prothrombin levels and a higher risk of venous thromboembolism. Although current evidence does not identify it as a direct common cause of LCPD, thrombophilia states in general may contribute to its pathogenesis [26,44].

The rs6025 variant of the *factor V* (*FV*) gene corresponds to *Factor V Leiden* (*FVL*). It involves a single-nucleotide substitution at position 1691 (1691G>A) in exon 10 of the *FV* gene, resulting in an arginine-to-glutamine change at position 506 (p.R506Q), which is one of the cleavage sites for protein C (PC). This alteration renders FV resistant to inactivation by activated protein C (APC), leading to a hypercoagulable state [45]. *FVL* has been significantly associated with LCPD in some North American and Dutch populations [28,29,30,31]. However, other publications have not found an association of *FVL* in Spanish and Serbian patients with this disease [26,32].

In various reports of Mexican patients with LCPD, an increase in the activity of factors V and IX, as well as elevated homocysteine (Hcy) concentrations, has been documented [25,27,33]. The A>G rs6048 (Malmö) SNV in the *factor IX* (*FIX*) gene has been associated with increased FIX activity, which could contribute to a higher risk of venous thromboembolism (VTE) [34]. However, in studies conducted in a Mexican population, this *FIX* SNV did not show an association with LCPD [25,33].

The *PAI-1* rs1799889 polymorphism (also known as the 4G/5G variant in the SERPINE1 gene) has been investigated in relation to LCPD in the context of prothrombotic states that could contribute to avascular necrosis of the FH. This variant has been associated with an increased risk of thrombosis due to reduced fibrinolytic capacity [34,35,36]. In a study of Mexican patients with LCPD, this SNV was evaluated; however, no statistically significant association with the disease was identified [25].

The methylenetetrahydrofolate reductase (MTHFR) and cystathionine β-synthase (CBS) enzymes regulate serum Hcy levels. The *MTHFR* rs1801133 (C677T) and *CBS* T833C rs115742905 SNVs are common variants in the *MTHFR* and *CBS* genes, respectively, and have been associated with reduced enzymatic activity. These alterations may lead to elevated Hcy levels and reduced folate availability, slightly increasing the risk of cardiovascular disease and stroke. In a study of Mexican patients with LCPD, these variants alone were not significantly associated with disease susceptibility; However, a significant association was observed for *MTHFR* rs1801133 after adjusting for elevated Hcy levels, particularly under a recessive inheritance model. The *MTHFR* rs1801133 variant alters folate metabolism and increases Hcy levels, which may promote endothelial damage and impair bone remodeling. Therefore, its association with LCPD may reflect disease susceptibility or severity rather than representing an independent primary risk factor [26,37,38].

Several reports have shown that the *β-fibrinogen* (*FGB*) gene rs1800790 (G455A) SNV is associated with elevated plasma fibrinogen levels, which may be further increased following exposure to tobacco smoke. This SNV has been proposed as a potential risk factor for LCPD, particularly in patients exposed to tobacco smoke [31,39]. These findings illustrate how hemostatic alterations, in conjunction with environmental factors, may contribute to the development of this disease. However, no significant association between *FGB* rs1800790 and LCPD has been demonstrated [25,33]. Several studies suggest that genetic variants involved in hemostatic regulation may play a central role in the etiology of LCPD, highlighting hemostasis as a potential therapeutic target (Table 3, Figure 4).

The available evidence supports the investigation of hemostatic alterations as potential contributors to the vascular mechanisms involved in LCPD. However, the reported genetic associations are heterogeneous across populations, and the causal contribution of individual variants remains uncertain. In particular, associations involving coagulation-related variants should be interpreted alongside phenotypic measures, including coagulation parameters and homocysteine concentrations, when available. Further studies are needed to determine whether these findings represent independent genetic effects, intermediate phenotypes, or interactions with other susceptibility factors.

### 4.2. Pro-Inflammatory States Axis

Several authors have investigated polymorphisms in genes involved in the pro-inflammatory system, highlighting that pro-inflammatory cytokines, interleukins (ILs), and related mediators may promote activation of the coagulation cascade by disrupting physiological anticoagulant mechanisms. Furthermore, inflammation plays a significant role in the pathological progression of LCPD by altering bone remodeling processes [25,35,46].

The *IL-3* rs31480 variant likely regulates cytokine expression and may influence hematopoiesis, inflammation, and microthrombosis. However, it is not an established causal factor in LCPD, but rather a possible biological modulator within a complex system. To date, it has not been associated with LCPD (Table 3) [32].

The *Interleukin-6* (*IL-6*) gene variants rs1800795 and rs1800796 are located within the promoter region of the *IL-6* gene and have been investigated in several inflammatory and vascular diseases. Their potential role in LCPD has also been explored; however, the available evidence remains limited and inconclusive, with reported associations ranging from weak to moderate. These variants do not appear to act as independent determinants of the disease, but rather as possible modulators of disease severity and progression [40,41].

In recent years, the pathophysiological role of the IL-23/IL-17 pathway in human diseases has been increasingly elucidated. The IL-23/Th17 axis, a key driver of chronic inflammation, is strongly associated with autoimmune disorders such as rheumatoid arthritis (RA) and psoriatic arthritis (PsA) [47,48].

Antigen-presenting cells (APCs), including dendritic cells, macrophages, and B lymphocytes, present antigens to CD4^+^ T cells, whose differentiation into Th17 cells is driven by a specific cytokine milieu, particularly IL-6, transforming growth factor beta (TGF-β), IL-1β, and IL-23. IL-23, a heterodimeric cytokine of the IL-12 family (p19/p40), signals through a receptor complex composed of IL-23R and IL-12Rβ1. Binding of IL-23 activates the JAK2/STAT3 pathway, leading to STAT3 phosphorylation, dimerization, and nuclear translocation, where it induces the transcription of pro-inflammatory genes. Th17 cells play a central role in inflammatory diseases by acting on synovial cells, T lymphocytes, and osteoclast precursors, promoting inflammation through the production of cytokines such as IL-17, IL-6, and TNF-α, as well as through activation of NF-κB signaling pathways. In summary, IL-23 drives the activation and expansion of Th17 lymphocytes, which secrete pro-inflammatory cytokines thereby enhancing neutrophil recruitment and sustaining chronic tissue inflammation (Figure 4).

Polymorphisms in the *IL-23R* gene (rs1569922, rs7518660 and rs7539625) may alter the expression or function of the IL-23 receptor. Depending on the allele, these variants can either enhance signaling, leading to an increased pro-inflammatory response, or attenuate it, resulting in reduced Th17 activation and a potential protective effect [25,48,49].

Additionally, IL-23 exerts both direct and indirect effects on bone metabolism by modulating osteoblast and osteoclast activity. Beyond its contribution to inflammation-mediated joint erosion, this cytokine may also play a role in the regulation of physiological bone remodeling. In this context, our research group has identified an association between IL-23R variants and susceptibility to LCPD. These findings suggest that inflammation plays a dual role in its etiopathogenesis, influencing both bone remodeling processes and hypofibrinolytic mechanisms [41,42,43].

As previously mentioned, the association between IL-6 gene variants and LCPD has been investigated in several studies. In Mexican patients with LCPD, the C allele and the homozygous mutant genotype (CC) of the *IL-6* rs1800795 polymorphism have been significantly associated with an increased risk of disease development [25,40,41]. This association is likely mediated by elevated IL-6 expression and enhanced inflammatory activity, which contribute to pathological bone necrosis and resorption of the FH. Similarly, a study conducted in Iranian children reported that the CC genotype is associated with an increased risk of LCPD. In contrast, findings regarding the *IL-6* rs1800796 variant remain inconsistent. In Mexican patients, no statistically significant association has been observed, whereas studies in Russian populations suggest that the GC genotype, or its interaction with other inflammatory factors, such as *TNF-α*, may be linked to an increased disease risk [25,40,50].

The *NOS3* gene encodes endothelial nitric oxide synthase (eNOS), an enzyme essential for the production of nitric oxide (NO), a potent vasodilator that regulates blood flow and exerts protective effects against inflammation and thrombosis [51]. The rs1799983 (G894T) variant in this gene has been investigated in relation to vascular pathologies, including susceptibility to avascular necrosis in children. Similarly, the rs2070744 (T786C) variant has been associated with reduced vasodilatory capacity and an increased risk of vascular diseases, as reported in studies of retinopathy and other conditions. Overall, the rs1799983 and rs2070744 SNVs in the *NOS3* gene may contribute to the development of LCPD by reducing NO production, thereby promoting endothelial dysfunction, chronic low-grade inflammation, and a prothrombotic state that may impair blood supply to the FH [42,43] (Table 3 and Figure 4).

Inflammatory pathways may represent an additional component of the proposed convergent framework. Associations involving IL6 and IL23R variants have been reported in selected populations, while findings for individual variants remain inconsistent. Although these pathways are biologically relevant to inflammation and bone remodeling, their specific contribution to LCPD susceptibility and progression requires further investigation, including functional studies and independent replication.

### 4.3. Metabolism–Bone Structure Axis

Bone structure and metabolism are linked through a dynamic bidirectional relationship in which cellular metabolic activity and bone architecture coordinately regulate tissue homeostasis and remodeling [52].

In LCPD, the involvement of structural genes is mainly linked to *COL2A1*, whereas *COL1A1* plays a more indirect role. The *COL1A1* gene encodes type I collagen, essential for bone strength; although its mutations are primarily associated with osteogenesis imperfecta, rare Perthes-like cases suggest that generalized bone fragility may predispose to avascular necrosis. However, current evidence, including studies in Mexican populations, does not support a significant association between *COL1A1* variants (rs1107946, rs2412298, rs1800012, rs2586498) and LCPD susceptibility. In contrast, *COL2A1*, located on chromosome 12q13.11, encodes type II collagen, the main structural component of articular and growth cartilage. Mutations in this gene, particularly glycine substitutions in the triple helix domain, produce unstable collagen fibers and are frequently associated with familial LCPD and FH osteonecrosis with autosomal dominant inheritance. These alterations may weaken the epiphyseal cartilage, increasing susceptibility to vascular compromise or mechanical collapse. Since the epiphyseal cartilage and subchondral bone are closely associated with the microvasculature of the femoral head, structural alterations in these tissues may contribute to vascular impairment and reduced blood supply. Furthermore, some cases diagnosed as LCPD may actually correspond to type II collagenopathies with similar radiological features. Variants such as rs121912891 and rs387106558 have been investigated as potential genetic markers; however, current evidence has not demonstrated a clear association with disease susceptibility [37,40].

Bone tissue is continuously remodeled through the concerted actions of bone cells, which include bone resorption by osteoclasts and bone formation by osteoblasts, whereas osteocytes act as mechanosensors and orchestrators of the bone remodeling process [40,52].

The relationship between these genetic variants and LCPD has been the subject of recent research aimed at elucidating the molecular basis of this avascular necrosis of the FH. Current evidence suggests that, although some of these markers are involved in inflammatory processes and bone remodeling, not all contribute equally to disease susceptibility. The RANK/RANKL/OPG pathway is the fundamental system that regulates bone remodeling, balancing bone formation and resorption [53,54]. RANKL (NF-κB Receptor Activator Ligand), produced by osteoblasts, activates the maturation and survival of osteoclasts and binds to RANK (NF-κB Receptor Activator). The RANKL-RANK binding initiates a signaling cascade for differentiation into active osteoclasts, promoting bone resorption. Meanwhile, OPG (Osteoprotegerin) is a protein secreted by osteoblasts that binds to RANKL, preventing it from binding to RANK and thus inhibiting bone resorption and preventing excessive bone destruction. The RANKL/OPG ratio determines the rate of bone remodeling. An imbalance with increased RANKL leads to bone loss. On the other hand, the activation of NF-κB results in the production of pro-inflammatory cytokines (such as IL-6, TNF-alpha) and chemokines, which promote inflammation and apoptosis of bone cells [55]. Studies in Mexican children have not shown a significant association between the *RANK* rs3018362 (G/A) and *RANKL* rs12585014 (G/A) polymorphisms and LCP. Although both genes are crucial for bone remodeling signaling, these specific SNVs do not appear to be determining risk factors on their own in this pathology. Furthermore, the *OPG* rs2073618 (C/G) polymorphism is a relevant high-risk marker detected in Mexican patients. It has been observed that carriers of the GC and GG genotypes have a more than four times greater risk of developing the disease [40] (Figure 4).

From another perspective, inflammation plays a key role in the pathological process of LCPD, as it significantly influences bone remodeling. Several studies have examined polymorphisms within the pro-inflammatory system, particularly in the *TNF-α* gene, which encodes a pro-inflammatory cytokine primarily produced by macrophages. TNF-α promotes inflammation, necrosis, and apoptosis, suppresses osteoblast activity, and stimulates the proliferation and differentiation of osteoclasts [56]. Elevated levels of TNF-α have been reported in patients with LCPD [56,57]. However, findings remain inconsistent; for instance, in populations studied in Iran and Serbia, there is no association between the *TNF-α* variants rs180062 and rs361525 and the disease [57] (Figure 4).

The bone metabolism and structural axis integrates genetic factors involved in extracellular matrix integrity and bone remodeling. Evidence concerning COL1A1, COL2A1, RANK, RANKL, and OPG suggests that these pathways warrant further investigation in LCPD. Nevertheless, the available findings do not support a uniform genetic effect across these genes. Associations involving OPG rs2073618 should be interpreted in relation to the characteristics of the study population and the statistical model used, while potential functional consequences require independent validation.

### 4.4. Environmental Factors

These factors contribute to the pathogenesis of LCPD. Variations in incidence by race, sex, and geographic region suggest that climate and sunlight exposure may influence susceptibility [58]. Higher prevalence in low socioeconomic populations highlights the role of nutritional deficiencies and environmental exposures [14]. Tobacco and wood smoke may promote endothelial dysfunction, hypoxia, and prothrombotic states [59,60]. Perinatal factors, such as low birth weight, and mechanical overload may further increase the risk of FH ischemia, contributing to disease development [14,61,62,63,64,65].

### 4.5. Epigenetics

Environmental factors influence epigenetics by modulating gene expression without altering the DNA sequence. These changes are dynamic and potentially reversible, depending on environmental exposures. Epigenetic mechanisms, such as DNA methylation, may contribute to the development of LCPD, as altered methylation patterns have been observed in affected patients [66].

MicroRNAs (miRNAs) are small non-coding RNAs that regulate gene expression and key cellular processes, including apoptosis and differentiation. Their dysregulation can contribute to disease. Overexpression of miR-214 has been associated with reduced chondrocyte viability in LCPD [67]. Additionally, exosomal miRNAs may promote endothelial dysfunction and osteoclastogenesis [68]. miR-206 and SOX9, both involved in gene regulation and chondrocyte function, have also been linked to LCPD and proposed as potential therapeutic targets [69]. Overall, epigenetic factors likely contribute to LCPD etiology, either alone or in combination with other factors. However, the extent to which these changes contribute to disease susceptibility, reflect disease progression, or represent downstream responses remains uncertain. Further studies are required to establish their biological and clinical relevance.

### 4.6. Genetic Syndromes and Disorders with Perthes-like or Related Skeletal Phenotypes

Multiple genetic variants identified in LCPD have also been associated with other syndromes, supporting a complex and multifactorial etiology (Table 3). For example, the A1226G mutation in the GBA gene, characteristic of Gaucher disease, has been reported in patients with LCPD and is associated with bone infarction, osteonecrosis, and skeletal abnormalities resembling those of the disease [70,71,72,73].

Ciliopathies such as Bardet–Biedl syndrome (BBS) further highlight this complexity. Caused by defects in primary cilium function, BBS presents with multisystem involvement, including skeletal abnormalities, and has been reported in association with LCPD in patients carrying BBS6/MKKS mutations [74,75,76,77]. Similarly, Floating–Harbor syndrome, caused by mutations in the SRCAP gene, suggests that altered transcriptional regulation may contribute to shared pathogenic pathways [78,79].

Collagen-related disorders reinforce the role of extracellular matrix alterations. Associations with Stickler syndrome (*COL2A1*) and osteogenesis imperfecta (*COL1A1*) suggest that defects in collagen structure may predispose to femoral head vulnerability and impaired bone remodeling [80,81].

Chromosomal abnormalities, particularly Down syndrome, have also been linked to LCPD, with a notable prevalence of hip disorders in this population [82]. Additionally, rare conditions such as Buschke–Ollendorff syndrome (*LEMD3* mutations) support the involvement of altered bone metabolism [22].

Vascular and metabolic disorders further contribute to this framework. Kawasaki disease underscores the role of vascular compromise in femoral head ischemia [22,83,84], while Morquio syndrome (GALNS mutations) highlights the impact of skeletal dysplasia and matrix dysfunction on disease development [85,86].

Collectively, these associations suggest that LCPD shares pathogenic mechanisms with diverse genetic disorders, involving ciliary dysfunction, extracellular matrix alterations, impaired bone metabolism, and vascular insufficiency.

**Table 3:** Some syndromes and mutations related to Legg–Calve–Perthes disease.

Disease	Mutation or Gene	Reference Number
Gaucher disease	1226G (N370S)	
Bardet–Biedl syndrome	MKKS/BBS6	[21,71]
Trichorhinophalangeal syndrome type I (TRPS1)	TRPS1	[87]
Floating–Harbor Syndrome (FHS)	SRCAP	[79]
Stickler syndrome	COLA2A1	[88]
Down’s syndrome	Chromosome 21	[82]
Buschke–Ollendorff syndrome	LEMD3	[22]
Kawasaki disease	ND	[84]
Morquio A syndrome	GALNS	[85]

ND: Not described.

### 4.7. Integration of the Three Axes and Other Factors

The evidence summarized in Table 2 supports the concept that LCPD is a complex multifactorial disorder resulting from the convergence of vascular, inflammatory, and bone remodeling mechanisms. Rather than being explained by a single genetic alteration, disease susceptibility appears to arise from the combined effects of multiple genetic variants together with environmental and epigenetic factors.

Most available studies have evaluated single-nucleotide variants independently. Consequently, the absence of significant associations for many SNVs should be interpreted cautiously, as individual variants may exert only modest effects that become biologically relevant through gene–gene (epistatic) and gene–environment interactions. Environmental exposures, including passive smoking, nutritional status, obesity, low birth weight, mechanical loading, vascular insults, and socioeconomic conditions, may further modify individual susceptibility and disease severity.

Within this framework, hypercoagulability may represent an early pathogenic event by promoting thrombotic occlusion of the epiphyseal microcirculation, resulting in ischemia and osteonecrosis of the femoral head. Subsequent activation of inflammatory pathways amplifies tissue injury through cytokines such as IL-6, TNF-α, and mediators of the IL-23/Th17 axis, which promote endothelial dysfunction, enhance prothrombotic activity, impair osteoblast function, and stimulate osteoclast-mediated bone resorption. Progressive alterations in bone metabolism and structural integrity, together with mechanical overload, ultimately contribute to femoral head collapse despite ongoing reparative processes such as angiogenesis and reossification.

This integrated model provides a biological explanation for the heterogeneous and frequently inconsistent genetic associations reported across studies. Rather than excluding a pathogenic role, negative findings may reflect the polygenic architecture of LCPD, small individual effect sizes, population heterogeneity, and methodological differences among studies.

Several limitations should be acknowledged. Most available studies were observational case–control genetic association studies with relatively small sample sizes, limiting statistical power and external validity. Considerable heterogeneity existed regarding study populations, genetic markers, outcome definitions, and analytical approaches. Furthermore, functional validation of many candidate variants remains scarce, and replication in independent populations is still limited.

Given the rarity of LCPD and the limited number of published genetic association studies, Pub-Med/MEDLINE was considered the primary source of peer-reviewed biomedical literature for this review. Although searches of additional databases might have identified further relevant records, their omission represents a limitation of the present review.

Despite these limitations, the present review provides an updated and biologically integrated overview of the genetic architecture of LCPD. Unlike previous reviews, which primarily summarized the etiology and clinical aspects of the disease, this review organizes the available genetic evidence into three interconnected pathophysiological axes: hypercoagulability; inflammation; and bone metabolism/remodeling. This integrative framework not only updates the current evidence but also offers a mechanistic perspective that may facilitate future biomarker discovery, risk stratification, and the development of targeted therapeutic strategies.

The integration of the available evidence supports a multifactorial framework in which vascular, inflammatory, and bone remodeling mechanisms may interact in LCPD. However, most genetic studies have evaluated individual variants rather than integrated genetic networks. Consequently, the proposed convergence should be interpreted as a mechanistic hypothesis supported by biological plausibility and partially overlapping evidence, rather than as an established shared genetic architecture. Future research should investigate gene–gene and gene–environment interactions using adequately powered and independently replicated study designs.

## 5. Conclusions

In conclusion, the available evidence supports a multifactorial framework for LCPD involving potentially interacting vascular, inflammatory, bone remodeling, environmental, and epigenetic mechanisms. The proposed convergent framework provides a conceptual basis for organizing current findings and identifying research priorities. However, the evidence remains heterogeneous, and a shared genetic architecture has not been established. Future investigations should prioritize independent replication, functional validation, standardized phenotyping, and the evaluation of interactions among genetic and environmental factors, which could facilitate the identification of novel therapeutic targets aimed at modulating coagulation, inflammation, and bone remodeling processes.

## Figures and Tables

**Figure 1:**
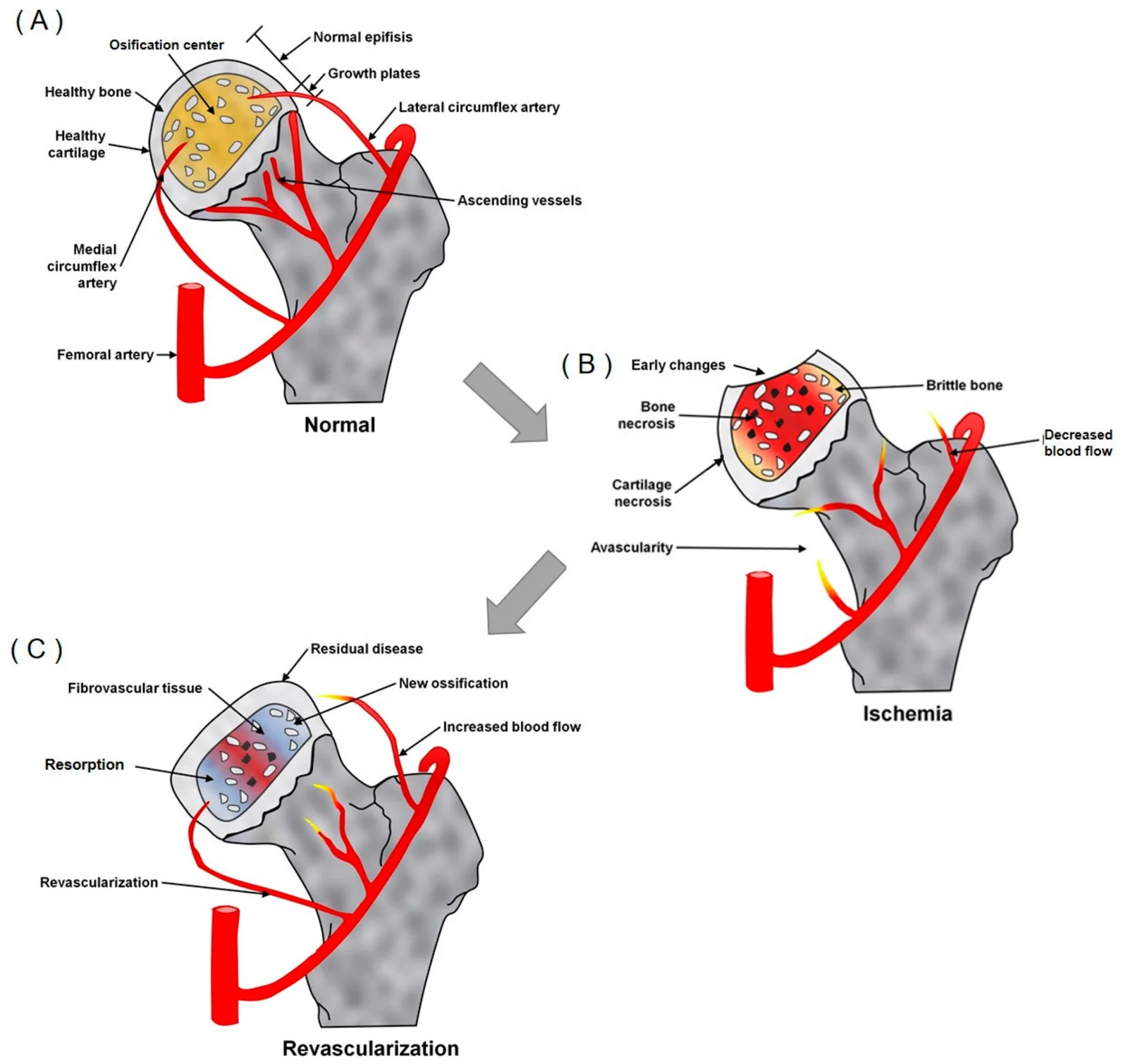
Pathogenesis of Legg–Calvé–Perthes disease (LCPD). (**A**) Normal stage: In the early stage of LCPD, the femoral head (FH) may appear radiographically normal or exhibit a small sclerotic epiphysis with widening of the joint space. (**B**) Ischemic stage: Interruption of the blood supply to the femoral epiphysis results in ischemic avascular osteonecrosis, leading to the death of osteocytes and other bone cells. Increased bone resorption, delayed bone formation, and continued mechanical loading of the hip contribute to progressive collapse and deformity of the FH. This stage typically lasts approximately 6 months. (**C**) Revascularization stage: Blood flow is restored through neovascularization and recanalization of pre-existing vessels. Necrotic bone is progressively resorbed and replaced by newly formed bone, promoting structural repair of the femoral head. This reparative phase may persist for more than 5 years.

**Figure 2:**
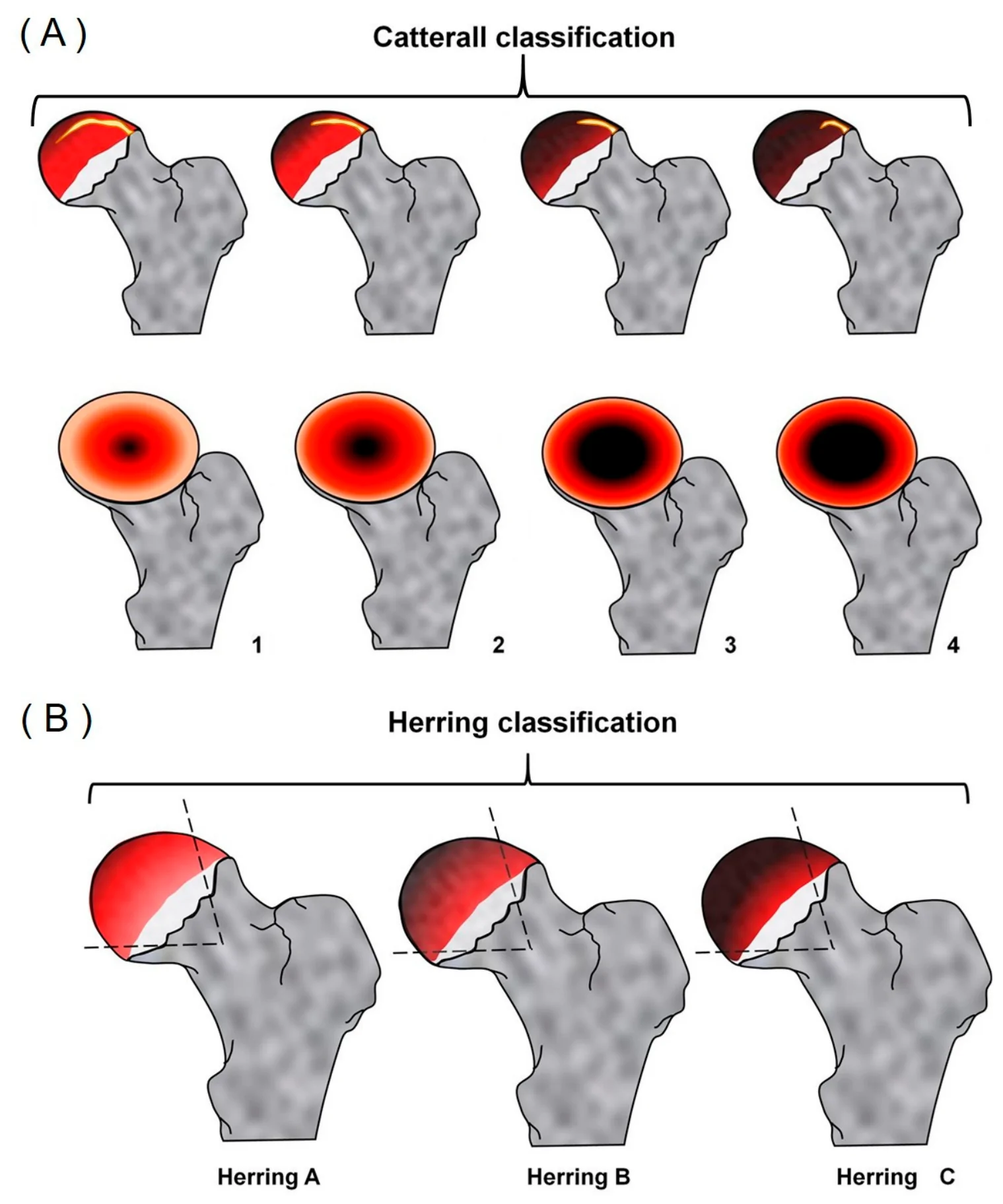
Classification systems for Legg–Calvé–Perthes disease (LCPD). The necrotic region is shown in red, whereas the structurally affected region is shown in black. (**A**) Catterall classification: This system classifies LCPD according to the extent of femoral head (FH) involvement, based on the approximate proportion of the epiphysis affected, and is used to estimate disease severity and prognosis. (**B**) Herring (lateral pillar) classification: This system categorizes LCPD according to the height of the lateral pillar of the femoral head. In Herring A, the lateral pillar height is preserved; in Herring B, more than 50% of the lateral pillar height is maintained; and in Herring C, less than 50% of the lateral pillar height remains.

**Figure 3:**
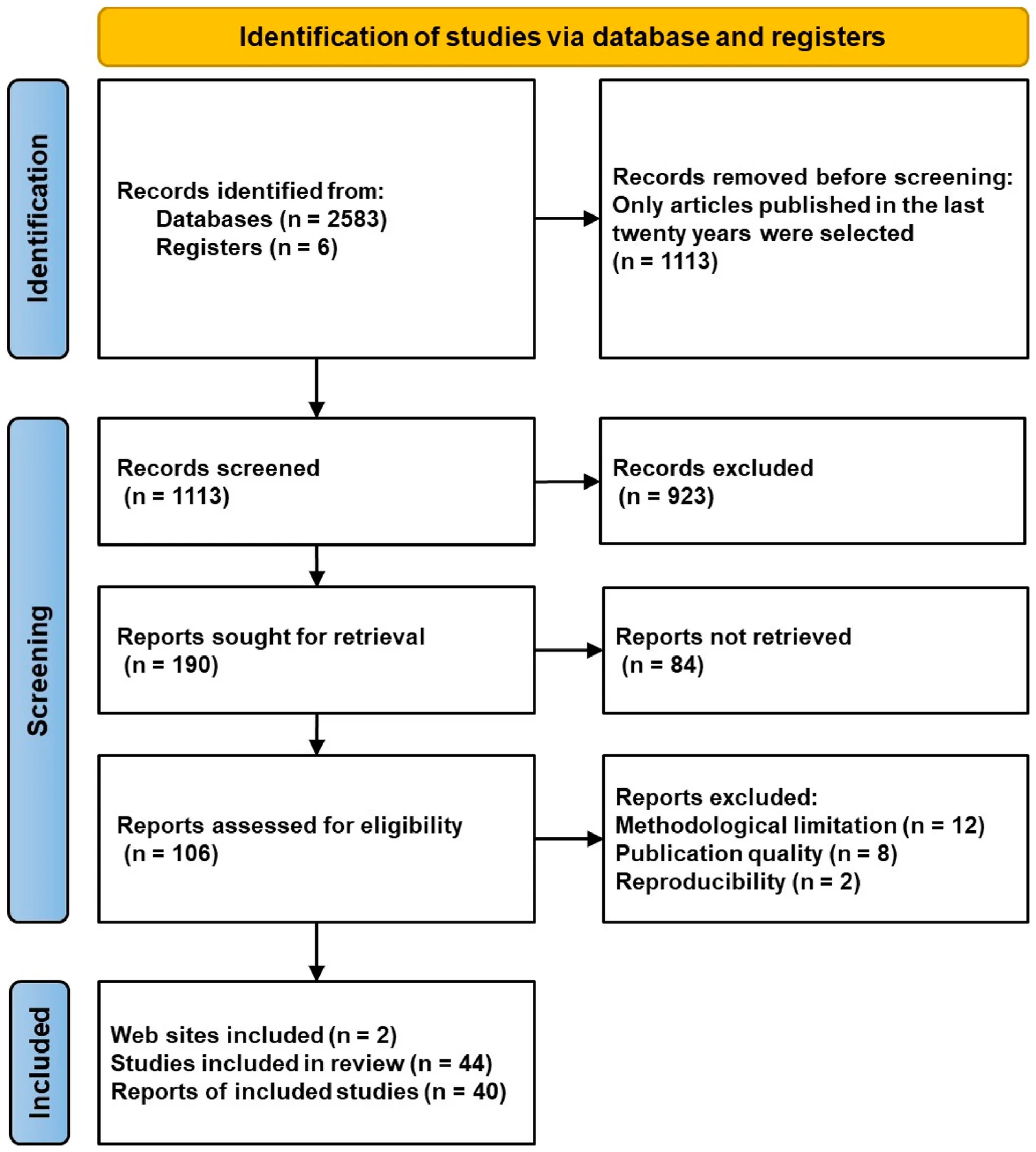
PRISMA flow diagram. PRISMA 2020 flow diagram illustrating the study selection process for the scoping review.

**Figure 4:**
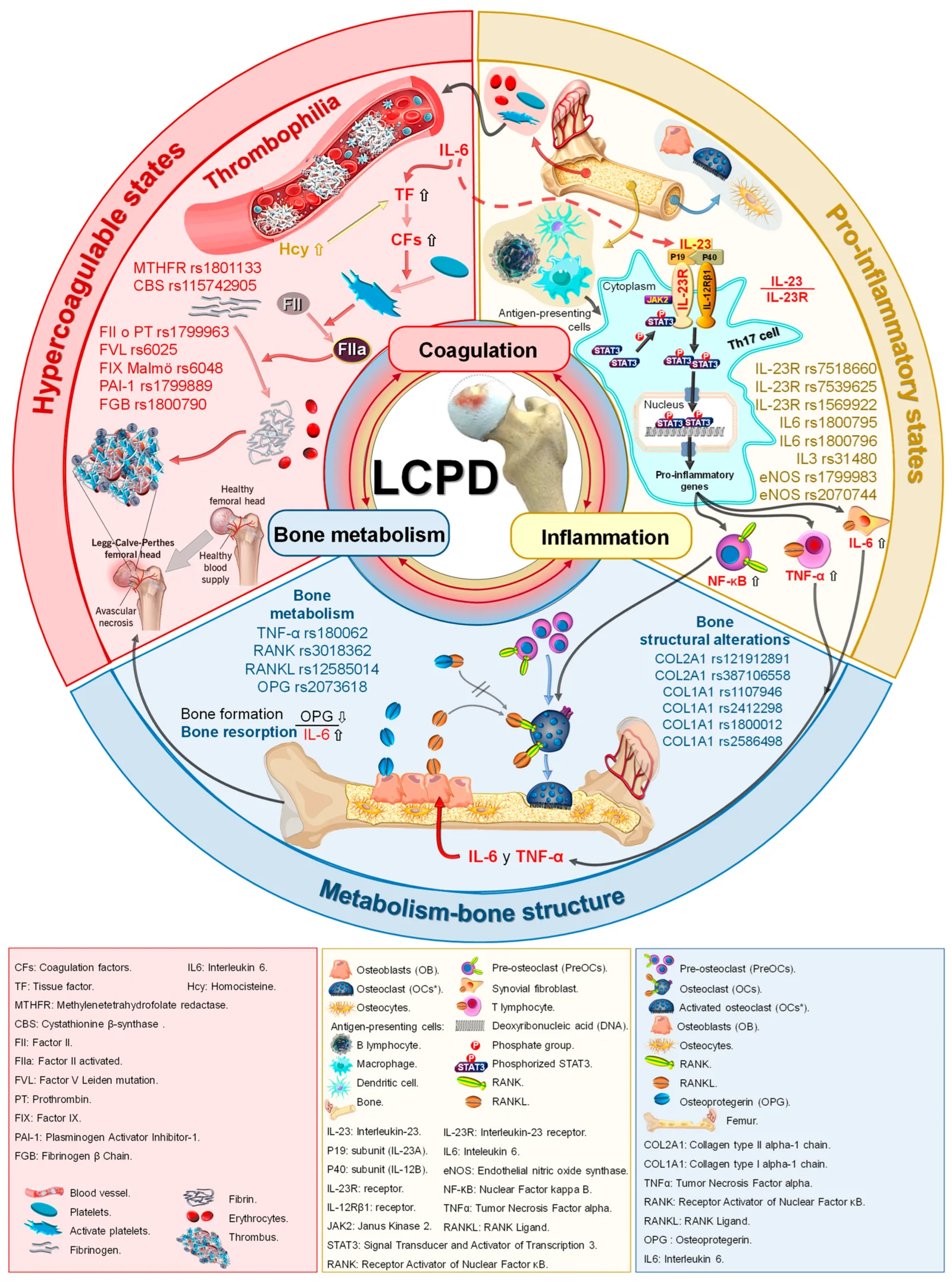
Proposed convergent molecular framework for Legg–Calvé–Perthes disease (LCPD). The framework integrates three interconnected biological axes: hypercoagulability, pro-inflammatory responses, and bone metabolism and remodeling. Genetic and epigenetic factors, together with environmental influences, are represented as potential modulators of these pathways. The proposed interactions are intended as a conceptual framework for interpreting the available evidence and generating mechanistic hypotheses; they do not imply that causal relationships have been established for all components.

**Table 1:** Eligibility framework based on the Population–Concept–Context (PCC) model.

PCC Element	Operational Definition
Population	Individuals with Legg–Calvé–Perthes disease (LCPD) and relevant preclinical models, including animal, cellular, in vitro, ex vivo, or patient-derived biological models.
Concept	Genetic and epigenetic factors and molecular pathways potentially involved in disease susceptibility or pathogenesis, including genetic variants, polymorphisms, mutations, gene expression, DNA methylation, microRNAs, inflammatory and coagulation-related mechanisms, vascular pathways, apoptosis, angiogenesis, and bone metabolism and remodeling.
Context	Studies addressing the etiology, susceptibility, or pathogenesis of LCPD, including vascular, thrombotic, inflammatory, metabolic, structural, epigenetic, and syndromic mechanisms.

**Table 2:** Genes and single-nucleotide variants (SNVs) reported in Legg–Calvé–Perthes disease patients.

Axis	Gene	Symbol HGNC	rsID	Position/ Nomenclature	Association	References
Hypercoagulable State	CF II (Prothrombin)	FII (PT)	1799963	c.20210G>A	No	[26,27]
	CF V	FV	6025	c.1601G>A	Yes	[28,29,30,31]
					No	[26,32]
	CF IX	FIX	6048	c.580A>G	No	[27,33]
	Plasminogen activator inhibitor-1 (PAI-1)	SERPINE1	1799889	c.-675 4G/5G	No	[34,35,36]
	Methylenetetrahydrofolate reductase (MTHFR)	MTHFR	1801133	c.677C>T	Yes	[26,37]
					No	[38,39]
	Cystathionine beta-synthase (CBS)	CBS	115742905	c.833T>C	No	[37]
	Fibrinogen beta chain gene (FGB)	FGB	1800790	c.4577G>A	No	[25,33]
Pro-inflammatory State	Interleukin (IL) 3	IL-3	31480	c.162G>A	No	[32]
	IL-6	IL-6	1800795	c.174G>C	Yes	[40,41]
			1800796	c.572G>C	No	[40,41]
	Interleukin-23 receptor (IL-23R)	IL-23R	7539625	ncr	No	[25,33]
			1569922	ncr	Yes	[25,33]
			7518660	c.30G>A	No	[25,33]
	Endothelial nitric oxide synthase (*NOS3*)	NOS3	1799983	c.894G>T	Yes	[42,43]
			2070744	c.786T>C	Yes	[42,43]
Metabolism–bone structure State	Collagen (COL) type I alpha (A) 1 chain (COL1A1)	COL1A1	1107946	c.1997G/T	No	[37]
			2412298	c.1663indelT	No	[37]
			1800012	c.104-441G>T	No	[40]
			2586498	ncr	No	[40]
	COL type II A 1 chain (COL2A1)	COL2A1	121912891	c.2306G>A	No	[37]
			387106558	c.1888G>A	No	[37]
	Receptor Activator of Nuclear Factor kappa-B (RANK)	RANK	3018362	ncr	No	[40]
	*RANK* Ligand (RANKL)	RANKL	12585014	c.-1866G>A	No	[40]
	Osteoprotegerin (OPG)	OPG	2073618	c.9C>G	Yes	[40]
	Tumor necrosis factor alpha (TNF-α)	TNF-α	180062	c.-308 G>A	No	[40]

HGNC: HUGO Gene Nomenclature Committee. HUGO: Human Genome Organisation. rsID: Reference SNV cluster ID. CF: Coagulation factor. ncr: Is not located in a coding region. indel: insertion or deletion.

## Data Availability

No new data were created or analyzed in this study. Data sharing is not applicable to this article.
